# Virulence Characterization of *Listeria monocytogenes, Listeria innocua*, and *Listeria welshimeri* Isolated from Fish and Shrimp Using In Vivo Early Zebrafish Larvae Models and Molecular Study

**DOI:** 10.3390/pathogens9121028

**Published:** 2020-12-08

**Authors:** Arkadiusz Józef Zakrzewski, Wioleta Chajęcka-Wierzchowska, Anna Zadernowska, Piotr Podlasz

**Affiliations:** 1 Department of Industrial and Food Microbiology, University of Warmia and Mazury, Plac Cieszyński 1, 10-726 Olsztyn, Poland; wioleta.chajecka@uwm.edu.pl (W.C.-W.); anna.zadernowska@uwm.edu.pl (A.Z.); 2 Department of Pathophysiology Forensic Veterinary Medicine and Administration, University of Warmia and Mazury, ul. Oczapowskiego 14, 10-719 Olsztyn, Poland; piotr.podlasz@uwm.edu.pl

**Keywords:** *Listeria* spp., *Listeria monocytogenes*, *Listeria innocua*, *Listeria welshimeri*, *Danio rerio*, pathogenicity

## Abstract

Listeriosis is one of the most notable foodborne diseases and is characterized by high rates of mortality. *L. monocytogenes* is the main cause of human listeriosis outbreaks, however, there are isolated cases of disease caused by other species of the genus *Listeria*. The aim of this study was to evaluate strains of *L. monocytogenes* (*n* = 7), *L. innocua* (*n* = 6), and *L. welshimeri* (*n* = 2) isolated from fish and shrimps for their virulence based on the presence of virulence genes and the in vivo *Danio rerio* (zebrafish) larvae models. A total of 15 strains were analyzed. The zebrafish larvae model showed that the larvae injected with *L. monocytogenes* strains were characterized by the lowest survival rate (46.5%), followed by *L. innocua* strains (64.2%) and *L. welshimeri* (83.0%) strains. Multiplex PCRs were used for detection of selected virulence genes (*lux*S, *act*A2, *prf*A, *inl*B, *rrn, iap*, *sig*B, *plc*B, *act*A, *hly*A), the majority of which were present in *L. monocytogenes*. Only a few virulence-related genes were found in *L. welshimeri*, however, no correlation between the occurrence of these genes and larval survival was confirmed. This research highlights the importance of the potential impact that *Listeria* spp. strains isolated from fish and shrimps may have on consumers.

## 1. Introduction

Species *L. monocytogenes*, *L. ivanovii*, and *L. seeligeri* are considered to be the main causes of listeriosis in animals and humans, however, due to the occurrence of single cases of infection caused by other species, including *L. innocua*, it seems necessary to study the virulent potential among others species of the genus *Listeria* [1].

The genus *Listeria* currently includes seventeen species [2]. For humans, *L. monocytogenes* is most often involved in foodborne outbreaks of listeriosis [3]. The other two species mentioned above are rarely reported as causes of human listeriosis [4,5,6], though there are reports of *L. welshimeri* being isolated from human fecal samples [7].

Listeriosis can develop as gastroenteritis with fever or present a more severe course with meningitis and sepsis. Parenteral symptoms are primarily found in people at risk, including pregnant women, newborns, the elderly, and people with reduced immunity (transplant recipients, patients receiving anti-cancer drugs or undergoing immunosuppressive therapy, or Acquired immunodeficiency syndrome (AIDS) patients). In this group, listeriosis has a mortality rate of 20–30% [3].

*L. monocytogenes* has a sophisticated intracellular lifecycle and pathogenic strategy. Research shows that the pathogenicity island 1 is responsible for this strategy. Pathogenicity island 1 (LIPI-1) contains the *prf*A-cluster. The *prf*A gene is a transcription activator that regulates the expression of various *L. monocytogenes* virulence genes, and *plc*A is a phospholipase C with phosphatidylinositol activity. LIPI-1 also contains a lecithinase operon, which contains genes whose presence was investigated in the study, including *act*A and *plc*B. *act*A gene coding for actin polymerizing protein, which recruits and polymerizes actin filaments to *Listeria* when it is in the cell to promote its intracellular motility. *plc*B gene encodes phospholipase C with lecithinase activity and helps *Listeria* to escape from phagosomes, thus promoting the spread of *Listeria* to other cells. LIPI-1 also contains *hly*, which encodes listeriolysin O (LLO), which lyses erythrocytes and other cells, but also lyses vacuoles of eukaryotic cells, allowing *Listeria* to spread through the cytoplasm. Removal of the *hly* gene leads to loss of virulence, which proves the importance of LLO in the pathogenesis of *L. monocytogenes* [8].

The aim of the study was to evaluate strains of *L. monocytogenes, L. innocua*, and *L. welshimeri* isolated from fish and shrimp for their virulence based on the presence of virulence genes and the in vivo zebrafish larvae models. 

## 2. Results

**Figure 1:**
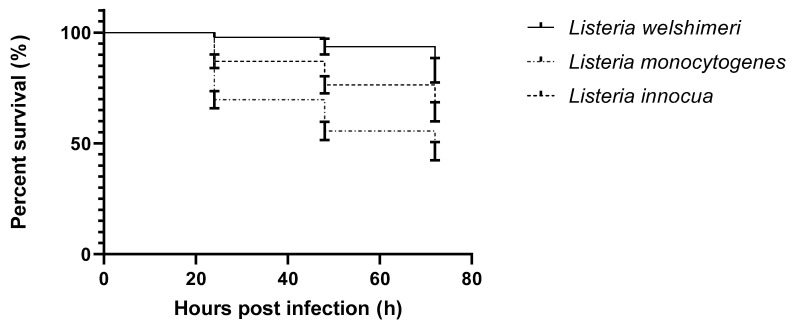
Survival curves of early zebrafish larvae injected with *L. monocytogenes*, *L. innocua*, and *L. welshimeri*.

The virulence of *L. monocytogenes* has been well characterized, however, most previous research has focused on clinical strains, whereas there may be large variations among species and environmental strains [9]. We tested the outcome of experimental infection of early zebrafish larvae with *Listeria monocytogenes*, *Listeria innocua*, and *Listeria welshimeri*. The average survival curves are presented in Figure 1.

The lowest survival, after 72 h, among zebrafish larvae can be observed when challenged with *L. monocytogenes* strains (46.5%). The average survival of *L. innocua*-infected larvae is higher and reached 64.2%; the highest survival rate was found in *L. welshimeri*-infected larvae, which was 83.0%. The differences between the species differ significantly (*p* value < 0.0001) (Figure 1).

**Figure 2:**
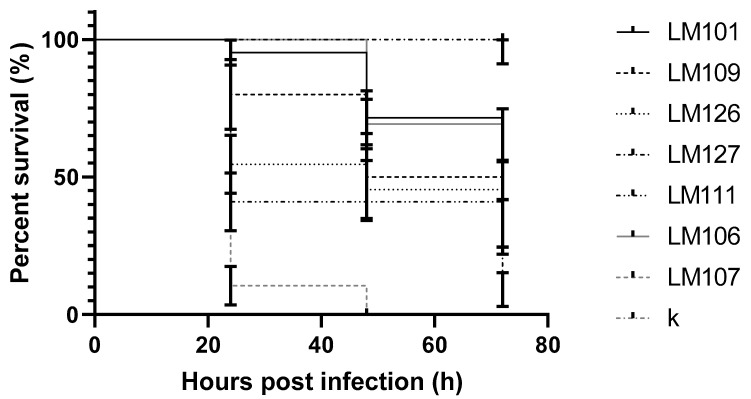
Survival curves of early zebrafish larvae injected with *L. monocytogenes*; k: control; 101, 106, 107, 109, 111 126, 127: strains.

The survival rate of larvae infected with *L. monocytogenes* differed statistically significantly (*p* value < 0.0001) between the strains. After 72 h of the experiment, it was observed that the survival rate varied from 0.0% to 95.5%. In the case of one strain (LM107), none of the infected larvae survived after two days of the experiment (Figure 2).

**Figure 3:**
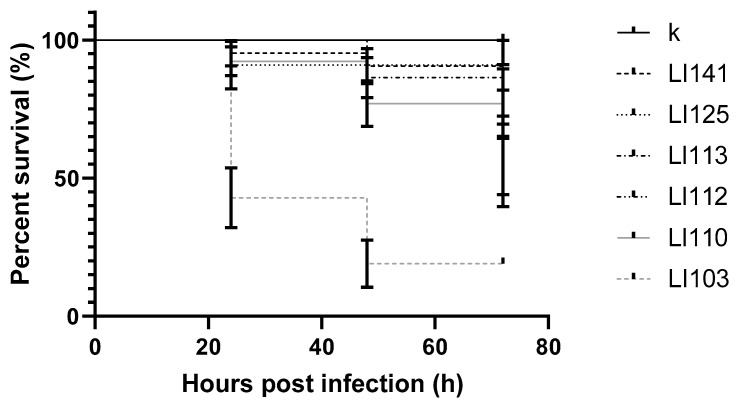
Survival curves of early zebrafish larvae injected with *L. innocua;* k: control; 103, 110, 112, 113, 125, 141: strains.

As with *L. monocytogenes*, survival among larvae infected by *L. innocua* also varied widely between strains. For the least virulent strain, the survival rate was 95.4%, while for the most virulent strain, it was 19.0%. The differences between the strains were statistically significant (*p* value < 0.0001). Survival results for individual strains are presented in Figure 3.

**Figure 4:**
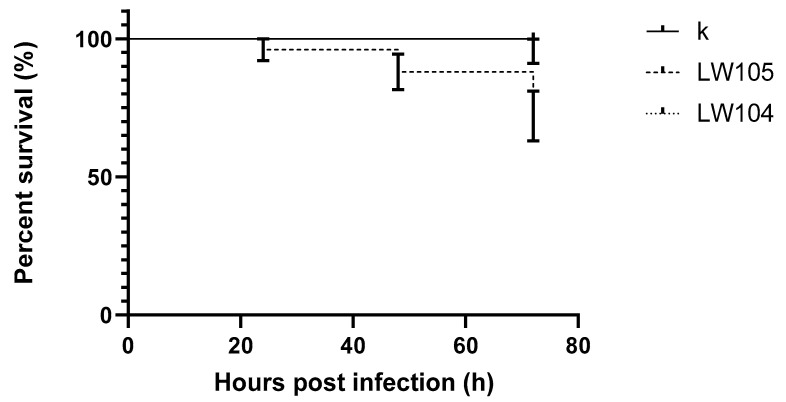
Survival curves of early zebrafish larvae injected with *L. welshimeri;* k: control; 105, 104: strains.

Of the two *L. welshimeri* strains, LW104 showed no virulence, and all infected larvae survived the 72-h experiment. For the second strain (LW105), larvae survival was 72.0%. These differences are statistically significant (*p* value = 0.0107). Survival results for individual strains are presented in Figure 4.

**Figure 5:**
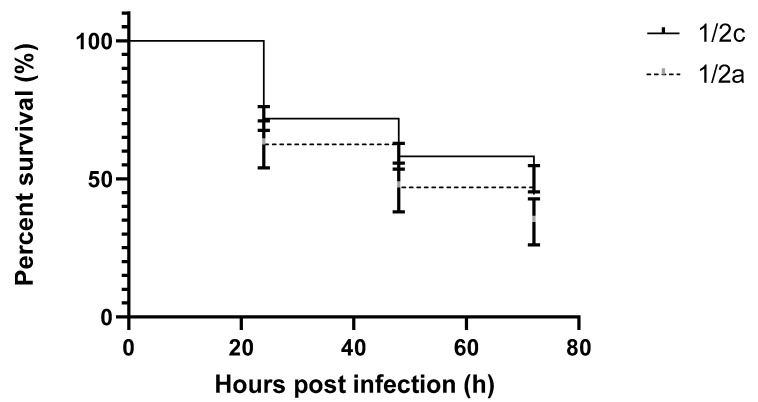
Survival curves of early zebrafish larvae injected with *L. monocytogenes;* serotype 1/2a (filled, black line), serotype 1/2c (dotted, black line).

Among the tested strains of *L. monocytogenes* there were two serotypes: 1/2a and 1/2c. The results of average survival for each stereotype are presented in Figure 5. These differences are not large: for serotype 1/2a the average survival was 46.9%, while for serotype 1/2c the average survival was 58.2%; these differences are not statistically significant (*p* value = 0.1205).

The frequencies of the virulence genes identified in *Listeria* spp. strains are listed in Table 1.

Nine virulence profiles were characterized in the study. The most common profile was *plc*B-*act*A2-*hly*A-*sig*B-*act*A-*prf*A-*inl*B-*rrn-iap-lux*S (26.7%), the next most common profiles were *sig*B-*rrn* (20.0%) and *plc*B-*hly*A-*sig*B-*act*A-*prf*A-inlB-rrn-iap (13.3%). 

The study examined the correlation between the occurrence of genes associated with virulence and the survival of zebrafish larvae. The strongest negative correlation was observed for the *actA* (−0.3191) and *hly* (−0.2876) genes. The next genes with negative correlation were the *prf*A and *iap* genes, whose values were both −0.1817. A positive correlation was observed for two genes, 0.2648 for the *lux*S gene and 0.1953 for the *rrn* gene. These results are not statistically significant (*p* > 0.005 for each gene).

## 3. Discussion

The latest European Food Safety Authority (EFSA) data shows that almost half (41.7%) of the Rapid Alert System for Food and Feed notifications for *L. monocytogenes* in 2008–2016 concerned fish and their products [10]. It is *L. monocytogenes* that is considered the main threat to humans among species of the genus *Listeria*, while there have been rare cases of infection with other species of this genus [11]. Pathogenicity is closely related to the *prf*A-virulence gene cluster (*p*VGC), containing the *prf*A, *plc*A, *hly*, *mpl*, *act*A, and *plc*B genes, although there are other genes that are indirectly treated as virulence factors [12].

In the present study, the intraspecies variations in the virulence profiles were investigated. Research suggests that the *hly* gene is crucial for the virulence of *L. monocytogenes* strains [12,13]. In the present study, the *hly* gene was present in each *L. monocytogenes* strain for both serotype 1/2a and 1/2c. Additionally, it was found in one atypical strain of *L. innocua*. The atypical *L. innocua* strain was characterized by very high survival among larvae (80.9%), while the most virulent strain 103 had only the *sig*B and *rrn* genes. The most likely explanation seems to be the inactive form of the genes from prfA-cluster, while the high mortality rate of larvae infected with strain 103 may be due to genes other than those tested in this experiment. Cases of such strains have already been reported [14,15]. There is also a study indicating other species of the genus *Listeria* possessing the *hly* gene, including *L. seeligeri* [16]. In the study of the frequency of genes from *p*VGC cluster, they were found in each of the *L. monocytogenes* strains. Similar results were obtained by other researchers in the study of food products [17,18,19]. According to Orsi et al. (2016), *L. welshimeri* possessing a *p*VGC cluster have not been confirmed [2]. Due to possible polymorphism in the *act*A gene, two sets of primers, *actA*1 and *actA*2, were used [20]. For one strain, the *act*A gene was detected by the sequence for *actA*2 primer and was not detected for the second set of primers. In the case of genes unrelated to the *p*VGC cluster, they most often occurred in *L. monocytogenes* and *L. innocua*. Only two of ten genes (*sig*B and *rrn*) occurred in *L. welshimeri*.

In this work, a model for infection was established in zebrafish larvae in order to study the pathogenesis of *Listeria* sp. In vivo research on zebrafish larvae has also been used to study the virulence of various pathogens including *Escherichia coli* [21], *Staphylococcus aureus* [22], and *Aeromonas hydrophila* [23] but also fungal or viral agents. As for the use of zebrafish larvae to study the pathogenicity of *Listeria*, only *L. monocytogenes* has been used in previous research [24]. Until now, in vivo models using *Danio rerio* to study the virulence of *L. monocytogenes* focused on the model itself and not on testing strains from environmental samples. One study from 1996 investigated *Listeria* spp. strains from food samples, however, experiments were carried out at 22 °C, a temperature that differs from the optimal temperature for the expression of *Listeria* virulence genes, therefore this study will not be considered in the discussion [25].

In this study, the pathogenicity of *L. welshimeri* and *L. innocua* strains isolated from raw fish and shrimp was investigated. *L. monocytogenes* strains were characterized by their significant virulence potential. Mortality among zebrafish larvae was 65.6%. The study did not prove statistically significant correlation between the occurrence of known virulence genes and virulence in an in vivo model. 

Comparing the virulence of *L. innocua* and *L. monocytogenes*, it can be concluded that *L. innocua* has been observed to be less virulent, however, mortality among larvae varied between 4.6% and 80.9%. Although this species is considered to be avirulent, there are individual data reporting patients with diseases caused by *L. innocua*. In 2014, *L. innocua* was the cause of meningitis. Researchers showed the presence of the *prf*A, *inl*A, *inl*B, *inl*C, *hly*, *plc*A, *plc*B, *mpl*, *iap*, *clp*E, *daa*A, and *act*A genes in the investigated strain [26]. The first fatal case caused by *L. innocua* occurred in 2003. The cause of death was bacteremia. In the cited study, the authors did not specify a virulence profile [27].

There is little data on *L. welshimeri* virulence. In our study, only two *L. welshimeri* strains were tested, none of which showed high mortality among *Danio rerio* larvae. A similar study on a live model, in this case mice, was carried out in 1986. The virulence of 16 *L. welshimeri* strains were investigated, each of which proved to be avirulent [28].

## 4. Materials and Methods 

### 4.1. Ethics Statement 

All fish were housed in the fish facility of Laboratory of Genomics and Transcriptomics in the University of Warmia and Mazury, Olsztyn, Poland, which was built according to local animal welfare standards. All animal procedures were performed in accordance with Polish and European Union animal welfare guidelines. According to the European Directive 2010/63/EU and Polish law regulations Dz.U. z 2015 r. poz. 266, all procedures performed in the present study including the use of early life-stages zebrafish and the euthanasia for the purpose of organ dissection did not require ethics committee permission.

### 4.2. Bacteria 

The different *Listeria* species and serotypes isolated from fish (*Salmo salar* and *Oncorhynchus mykiss*) and shrimp (*Penaeus monodon*) sources used in this study were obtained from the microbiological collection of the Department of Industrial and Food Microbiology University of Warmia and Mazury in Olsztyn and are listed in Table 2. Strains were grown from frozen stocks in 5 mL of Brain Heart Infusionbroth (Merck, Darmstadt, Germany) overnight at 37 °C. Subsequently, the strains were transferred to 5 mL of BHI broth and incubated for 24 h at 37 °C. After incubation, the strains were centrifuged and washed three times with sterile saline. Eventually, phenol red (Merck, Darmstadt, Germany) was added to obtain a 0.05% solution in order to be able to observe the inoculation process.

### 4.3. Pathogenicity Assay Using Zebrafish Larvae

All fifteen *Listeria* species strains were analyzed for virulence potential in the zebrafish (*Danio rerio*) larvae model, as described previously [29]. Zebrafish were kept and handled in compliance with the guidelines of the European Union for handling laboratory animals (http://ec.europa.eu/environment/chemicals/lab_animals/home_en.htm). Zebrafish studies were performed on wild type and on Tg(mCherry) 72 h post-fertilization. 

Larvae were microinjected in yolk with the different strains (ca. 50 bacteria per nL). For survival assays, embryos (*n* ≥ 15 per strain per experiment) were kept in 48-well plates and analyzed at regular time intervals for mortality (scored by the absence of heartbeat). Controls for the experiment were larvae inoculated with a 0.05% solution of phenol red (Merck, Darmstadt, Germany). Survival data are presented in Kaplan–Meier plots, and the significance of the data was analyzed with a log rank (Mantel–Cox) test using GraphPad Prism software version 8.0 (GRAPH PAD software Inc, San Diego, CA, USA).

### 4.4. PCR Assay

Total DNA was extracted from isolated strains according to the instruction manuals of commercial DNA extraction kits (A&A Biotechnology, Gdynia Poland). The presence of the virulence-associated genes were detected by PCR in three reactions (Table 3). PCR amplification was performed using 2 μL of template DNA, 2 μL of each primer (100 pmol), and 12.5 μL of 2 × DreamTaq PCR Master Mix (Thermo Fisher Scientific, Waltham, MA, USA) in a total reaction volume of 25 μL. The PCR conditions consisted of an initial denaturation step at 94 °C for 3 min, followed by 35 cycles of denaturation at 94 °C for 30 s, annealing for 30 s, and elongation at 72 °C for 1 min. A final extension step was carried out at 72 °C for 5 min. Correlations between gene occurrence and *Danio rerio* larval survival were calculated using Pearson correlation because the occurrence of genes was marked as 1 when the gene was present and 0 when it was absent, in which case the results of the Pearson correlation are identical to point-biserial correlation. 

## 5. Conclusions

The data presented in this study show that the diversity according to the virulence level of *Listeria* sp. strains isolated from fish and shrimp is complex and based on different mechanisms that seem to differ according to the species of the strains. In addition, as demonstrated by the experiment carried out in vivo, although *L. innocua* is not considered dangerous to human life, it may pose a potential threat.

## Figures and Tables

**Table 1:** The frequency of virulence-associated genes among *Listeria* spp. strains.

Prevalence of Virulence-Associated Genes (%)					
Genes	*L. monocytogenes*		*L. innocua* (*n* = 6)	*L. welshimeri* (*n* = 2)	Total
	1/2a (*n* = 2)	1/2c (*n* = 5)			
*plc*B	100.0	100.0	33.3	0.0	60.0
*ac*tA2	50.0	100.0	0.0	0.0	40.0
*hly*A	100.0	100.0	16.7	0.0	53.3
*sig*B	100.0	100.0	100.0	100.0	100.0
*act*A	100.0	100.0	33.3	0.0	60.0
*prf*A	100.0	100.0	0.0	0.0	46.7
*inl*B	100.0	80.0	0.0	0.0	40.0
*rrn*	100.0	80.0	66.7	100.0	80.0
*iap*	100.0	100.0	0.0	0.0	40.0
*lux*S	0.0	80.0	50.0	0.0	46.7

**Table 2:** Characteristics of the strains used in the research.

No.	Strain	Species	Serotype	Source
1	LW104	*L welshimeri*	nd.	*Salmo salar*
2	LW105	*L welshimeri*	nd.	*Salmo salar*
3	LI141	*L. innocua*	nd.	*Oncorhynchus mykiss*
4	LI125	*L. innocua*	nd.	*Oncorhynchus mykiss*
5	LI113	*L. innocua*	nd.	*Salmo salar*
6	LI112	*L. innocua*	nd.	*Salmo salar*
7	LI110	*L. innocua*	nd.	*Salmo salar*
8	LI103	*L. innocua*	nd.	*Salmo salar*
9	LM101	*L. monocytogenes*	1/2c	*Salmo salar*
10	LM109	*L. monocytogenes*	1/2a	*Salmo salar*
11	LM126	*L. monocytogenes*	1/2a	*Oncorhynchus mykiss*
12	LM127	*L. monocytogenes*	1/2c	*Salmo salar*
13	LM111	*L. monocytogenes*	1/2c	*Salmo salar*
14	LM106	*L. monocytogenes*	1/2c	*Penaeus monodon*
15	LM107	*L. monocytogenes*	1/2c	*Salmo salar*

nd.: not detected.

**Table 3:** List of primers used in research [30].

No.	Primers	Sequence (5′–3′)		Size (bp)	Target
1	luxS	F:	ATGGCAGAAAAAATGAATGTAGAAA	500	*lux*S
		R:	TTATTCACCAAACACATTTTTCCA		
	actA2	F:	GACGAAAATCCCGAAGTGAA	385	*act*A2
		R:	CTAGCGAAGGTGCTGTTTCC		
	prfA	F:	GATACAGAAACATCGGTTGGC	280	*prf*A
		R:	GTGTAATCTTGATGCCATCAGG		
	inlB	F:	AAAGCACGATTTCATGGGAG	148	*inl*B
		R:	ACATAGCCTTGTTTGGTCGG		
	rrn	F:	CAG CAG CCG CGG TAA TAC	938	*rrn*
		R:	CTC CAT AAA GGT GAC CCT		
	iap	F:	CAAACTGCTAACACAGCTACT	660	*iap*
		R:	TTATACGCGACCGAAGCCAAC		
2	sigB	F:	TCATCGGTGTCACGGAAGAA	310	*sig*B
		R:	TGACGTTGGATTCTAGACAC		
3	plcB	F:	CTGCTTGAGCGTTCATGTCTCATCCCCC	1150	*plc*B
		R:	ATGGGTTTCACTCTCCTTCTAC		
	actA	F:	CGCCGCGGAAATTAAAAAAAGA	839	*act*A
		R:	ACGAAGGAACCGGGCTGCTAG		
	hlyA	F:	GCAGTTGCAAGCGCTTGGAGTGAA	456	*hly*A
		R:	GCAACGTATCCTCCAGAGTGATCG		
